# A systematic analysis of FDA-approved anticancer drugs

**DOI:** 10.1186/s12918-017-0464-7

**Published:** 2017-10-03

**Authors:** Jingchun Sun, Qiang Wei, Yubo Zhou, Jingqi Wang, Qi Liu, Hua Xu

**Affiliations:** 1 School of Biomedical Informatics, The University of Texas Health Science Center at Houston, Houston, TX 77030 USA; 2 National Center for Drug Screening, Shanghai Institute of Materia Medica, Chinese Academy of Sciences, Shanghai, People’s Republic of China; 3 Department of Biomedical Informatics, Vanderbilt University, Nashville, TN 37203 USA

**Keywords:** Anticancer drugs, Drug-cancer network, Cancer-drug-target network, Drug repurposing

## Abstract

**Background:**

The discovery of novel anticancer drugs is critical for the pharmaceutical research and development, and patient treatment. Repurposing existing drugs that may have unanticipated effects as potential candidates is one way to meet this important goal. Systematic investigation of efficient anticancer drugs could provide valuable insights into trends in the discovery of anticancer drugs, which may contribute to the systematic discovery of new anticancer drugs.

**Results:**

In this study, we collected and analyzed 150 anticancer drugs approved by the US Food and Drug Administration (FDA). Based on drug mechanism of action, these agents are divided into two groups: 61 cytotoxic-based drugs and 89 target-based drugs. We found that in the recent years, the proportion of targeted agents tended to be increasing, and the targeted drugs tended to be delivered as signal drugs. For 89 target-based drugs, we collected 102 effect-mediating drug targets in the human genome and found that most targets located on the plasma membrane and most of them belonged to the enzyme, especially tyrosine kinase. From above 150 drugs, we built a drug-cancer network, which contained 183 nodes (150 drugs and 33 cancer types) and 248 drug-cancer associations. The network indicated that the cytotoxic drugs tended to be used to treat more cancer types than targeted drugs. From 89 targeted drugs, we built a cancer-drug-target network, which contained 214 nodes (23 cancer types, 89 drugs, and 102 targets) and 313 edges (118 drug-cancer associations and 195 drug-target associations). Starting from the network, we discovered 133 novel drug-cancer associations among 52 drugs and 16 cancer types by applying the common target-based approach. Most novel drug-cancer associations (116, 87%) are supported by at least one clinical trial study.

**Conclusions:**

In this study, we provided a comprehensive data source, including anticancer drugs and their targets and performed a detailed analysis in term of historical tendency and networks. Its application to identify novel drug-cancer associations demonstrated that the data collected in this study is promising to serve as a fundamental for anticancer drug repurposing and development.

## Background

In the last 50 years, numerous remarkable achievements have been made in the fight against cancer, starting from understanding cancer mechanisms to patient treatment. However, cancer remains as one of the leading causes of death in the world, which places a heavy burden on health services and society. Cancer involves abnormal cell growth with the potential to invade or spread to other parts of the body and encompasses more than 100 distinct diseases with diverse risk factors and epidemiology. Over the past five decades, scientific discoveries and technological advances, including modern molecular biology methods, high-throughput screening, structure-based drug design, combinatorial and parallel chemistry, and the sequencing of the human genomes have improved the drug discovery. However, the increasing cost of new drug development and decreasing number of truly efficient medicines approved by the US Food and Drug Administration (FDA) present unprecedented challenges for the pharmaceutical industry and patient healthcare, including the oncology [1, 2]. As the increasing availability of FDA-approved drugs and quantitative biological data from the human genome project, multiple strategies have been proposed to shorten the drug development process and significantly lower costs, including drug repurposing [3, 4] and network pharmacology [5, 6].

With advances in anticancer drug discovery and development in the last several decades, more than 100 anticancer drugs have been discovered and approved by the FDA [7, 8]. These drugs can be broadly classified into two basic categories: cytotoxic and targeted agents based on their mechanisms of action [9–11]. The cytotoxic agents can kill rapidly dividing cells by targeting components of the mitotic and/or DNA replication pathways. The targeted agents block the growth and spread of cancer through interacting with molecular targets that are involved in the pathways relevant to cancer growth, progression, and spread [12]. Those successful agents and their related data may provide valuable clues for further identification of novel drug targets, the discovery of novel anticancer drug combinations, drug repurposing, and computational pharmacology. Several reviews have provided the historical summary of these drugs, which revealed the trends of increasing proportion of targeted agents, particularly monoclonal antibodies [7, 8]. Recently network pharmacology has successfully applied in multiple fields such as target identification, prediction of side effects, and investigation of general patterns of drug actions [5, 13, 14]. Therefore, besides of updating the FDA-approved anticancer drugs, analysis of drug-disease/target networks will significantly increase our understanding of the molecular mechanisms underlying drug actions and provide valuable clues for drug discovery.

Thus, in this study, we first comprehensively collected the FDA-approved anticancer drugs by the end of 2014 and curated their related data, such as initial approval years, action mechanisms, indications, delivery methods, and targets from multiple data sources. According to their action mechanisms, we classified them into two groups: cytotoxic and targeted drugs. Then, we analyzed these data to reveal the different trends between the two groups. Besides, we analyzed the drug targets by investigating their subcellular locations, functional classifications, and genetic mutations. Finally, we generated anticancer drug-disease and drug-target networks to capture the common anticancer drugs across different types of cancer and to reveal how strongly the anticancer drugs and targets interact or drug-target networks. The network-assisted investigation provides us with novel insights into the relationships among anticancer drugs and disease or drugs and targets, which may provide valuable information for further understanding anticancer drugs and the development of more efficient treatments.

## Methods

### Collection of FDA-approved anticancer drugs and their relation information

We have collected anticancer drugs approved by FDA since 1949 to the end of 2014 from multiple data sources. We started the collection of the anticancer drugs from anticancer drug-focused websites, including National Cancer Institute (NCI) drug information [15], MediLexicon cancer drug list [16], and NavigatingCancer [17]. Then, we employed the tool MedEx-UIMA, a new natural language processing system, to retrieve the generic names for these drugs [18]. Using the generic names, we searched Drug@FDA [19] and downloaded their FDA labels. For those that cannot be found in the drugs@FDA, we obtained their labels from Dailymed [20] or DrugBank [21]. From the drug label, we manually retrieved the initial approval year, drug action mechanism, drug target, delivery method, and indication for each drug. We further checked the multiple sources such as the MyCancerGenome [22], DrugBank, and the several publications [4, 23] to obtain the drug targets. For drug category, we manually checked the ChemoCare [24] to assign the drugs as cytotoxic or targeted agents. In our curated drug list, we did not include the medicines to treat drug side effects, cancer pain, other conditions, or cancer prevention.

### Classes of drug targets and cancer

For these targeted agents, we collected their targets from FDA drug labels, DrugBank, and MyCancerGenome. We then manually curated the primary effect-mediating targets for each drug. We further retrieved the gene annotation from Ingenuity Pathway Analysis (IPA) [25] to obtain their subcellular location and family classes. For the indication, we first collected the detail information from FDA drug labels and then manually classified them into higher-level class for the purpose of data analysis. For example, drug idelalisib can be used to treat relapsed chronic lymphocytic leukemia (CLL), relapsed follicular B-cell non-Hodgkin lymphoma (FL), relapsed small lymphocytic lymphoma (SLL) from FDA labels. In our data analysis, we recorded the drug’s therapeutic classes as leukemia and lymphoma.

### Cancer genes and somatic mutations of the cancer genome

The cancer gene set contains 594 genes from the Cancer Gene Census, which have been implicated in tumorigenesis by experimental evidence in the literature (July 14, 2016) [26]. We obtained 50 oncogenes (OCGs) and 50 tumor suppressor genes (TSGs) with high confidence from Davioli et al. [27]. The somatic mutations were obtained from Supplementary Table 2 in one previous work [28]. The table contains the somatic mutations in 3268 patients across 12 types of cancer. They are bladder urothelial carcinoma (BLCA), breast adenocarcinoma (BRCA), colon and rectal adenocarcinoma (COAD/READ), glioblastoma (GBM), head and neck squamous cell carcinoma (HNSC), kidney renal clear cell carcinoma (KIRC), acute myeloid leukemia (LAML), lung adenocarcinoma (LUAD), lung squamous cell carcinoma (LUSC), ovarian cancer (OV), and uterine corpus endometrioid carcinoma (UCEC). The mutations include missense, silent, nonsense, splice site, readthrough, frameshift indels (insertions/deletions) and inframe indels [28].

### Network analysis

We built two networks based on our curated data, drug-cancer and drug-cancer-target networks. In the drug-cancer network, there are two types of nodes representing drug or cancer types and edges suggesting drug as the approved treatment for the cancer. In the drug-cancer-target network, there are three types of nodes representing cancer types, drug or drug target and edges indicating cancer-drug associations or drug-target interactions. The network degree is used to assess the toplogical feature of each cancer type and drug, i.e., the number of edges of each node in the network.

### Common target-based approach

We used common target-based approach to discover novel drug-cancer associations [29]. It is one of the “guilt-by-association” strategies based on the knowledge that whether the drugs shared common targets or not. If two drugs A and B have a common target, drug A is in current use for treating cancer type C and drug B is used for cancer type D, it is highly likely to be effective for drug A-cancer type D and drug B-cancer type C associations.

## Results and Discussion

### FDA-approved anticancer drugs

**Fig. 1:**
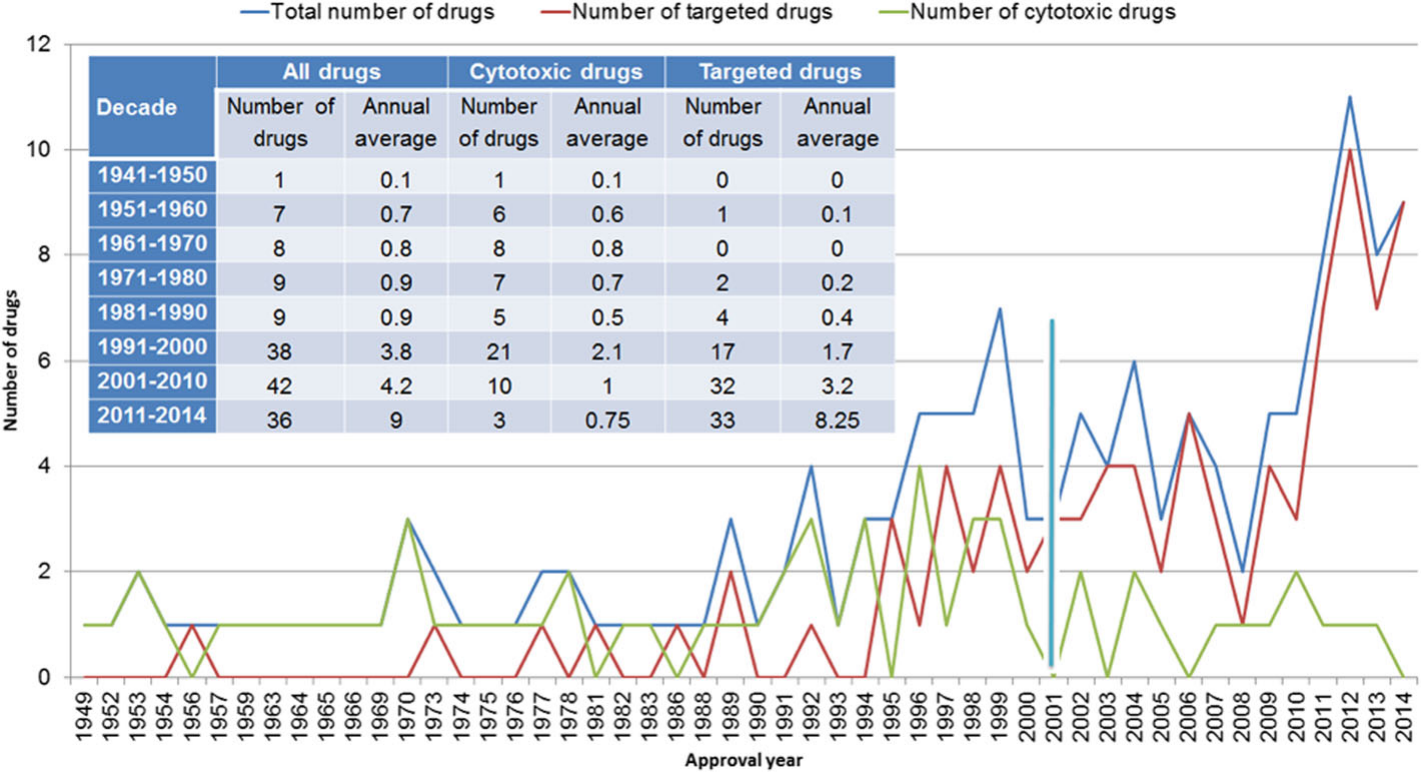
Number of anticancer drugs approved by FDA from 1949 to 2014. Approval dates were retrieved from FDA drug labels. Drugs were divided into two categories according to their action mechanisms. The inserted table is the summary of drug numbers for each decade

From 1949 to 2014, a total of 150 medicines has been approved with an indication for at least one type of cancer (Table 1). Notably, in this study, we did not include the drugs used to treat side effects of cancer treatment, cancer pain, and other conditions. Based on the mechanism of action (MOA), we grouped them into two groups: 61 cytotoxic drugs and 89 targeted drugs. Most of the cytotoxic drugs are alkylating agents, anti-microtubule agents, topoisomerase inhibitors while most of the targeted drugs belong to signal transduction inhibitors, gene expression modulators, apoptosis induces, hormone therapies, and monoclonal antibodies. Figure 1 shows that the number of approved drugs in cancer treatment had a gradual increase. In the later years (1991–2014), the number of approved anticancer (116 drugs) extremely increased compared to that of the previous five decades (1941–1990, 34 drugs). Even in the recent years (2011–2014), the annual average number was 9, which was about 2.5 times of that in 1991–2000 (3.8) or 2001–2010 (4.2). From 1991 to 2000, the number of anticancer targeted drugs (17) was similar to that of cytotoxic drugs (21). However, since the 2000s, the number of targeted drugs (65) was significantly higher than that of the cytotoxic drugs (13), which was about five times.

**Table 1:** Summary of FDA-approved anticancer drugs from 1949 to 2014

Drug	Approval year	Therapeutic class	Target gene	Delivery type
Cytotoxic				
Mechlorethamine	1949	Lung cancer; Leukemia; Lymphoma	DNA synthesis	Single
Leucovorin	1952	Colorectal cancer; Bone cancer	TYMS	Both
Methotrexate	1953	Leukemia; Breast cancer; Head and neck cancer; Lung cancer; Lymphoma; Bone cancer; Gestational trophoblastic disease	DHFR	Both
Mercaptopurine	1953	Leukemia	HPRT1	Combination
Busulfan	1954	Leukemia	DNA synthesis	Combination
Chlorambucil	1957	Leukemia; Lymphoma	DNA synthesis	Single
Cyclophosphamide	1959	Lymphoma; Multiple myeloma; Leukemia; Brain cancer; Ovarian cancer; Retinoblastoma; Breast cancer	DNA synthesis	Both
Vincristine sulfate	1963	Leukemia	TUBA4A; TUBB	Single
Dactinomycin	1964	Sarcoma; Gestational trophoblastic disease; Testicular cancer; Kidney cancer	RNA synthesis	Both
Vinblastine sulfate	1965	Lymphoma; Testicular cancer; Choriocarcinoma; Breast cancer	TUBA1A; TUBB; TUBD1; TUBE1; TUBG1	Combination
Thioguanine	1966	Leukemia	DNA synthesis	Combination
Procarbazine hydrochloride	1969	Lymphoma	DNA synthesis	Combination
Floxuridine	1970	Stomach cancer	DNA synthesis	Single
Fluorouracil	1970	Breast cancer; Colorectal cancer; Stomach cancer; Pancreatic cancer	DNA synthesis	Single
Mitotane	1970	Adrenal cortical carcinoma	Unknown	Single
Bleomycin	1973	Head and neck cancer; Lymphoma; Penile cancer; Cervical cancer; Vulvar cancer; Testicular cancer	DNA synthesis	Both
Doxorubicin hydrochloride	1974	Leukemia; Breast cancer; Stomach cancer; Lymphoma; Ovarian cancer; Lung cancer; Sarcoma; Thyroid cancer; Bladder cancer; Kidney cancer; Brain cancer	TOP2A; DNA synthesis	Single
Dacarbazine	1975	Melanoma; Lymphoma	DNA synthesis	Both
Lomustine	1976	Brain cancer; Lymphoma	DNA synthesis	Both
Carmustine	1977	Brain cancer; Lymphoma; Multiple myeloma	DNA synthesis	Both
Cisplatin	1978	Testicular cancer; Ovarian cancer; Bladder cancer	DNA synthesis	Both
Asparaginase	1978	Leukemia	Unknown	Combination
Streptozocin	1982	Pancreatic cancer	DNA synthesis; SLC2A2	Single
Etoposide	1983	Testicular cancer; Lung cancer	TOP2A; TOP2B	Combination
Ifosfamide	1988	Testicular cancer	DNA synthesis	Combination
Carboplatin	1989	Ovarian cancer	DNA synthesis	Both
Altretamine	1990	Ovarian cancer	DNA synthesis	Single
Fludarabine	1991	Leukemia	DNA synthesis	Single
Pentostatin	1991	Leukemia	ADA	Single
Paclitaxel	1992	Breast cancer; Lung cancer; Pancreatic cancer; Ovarian cancer; Sarcoma	TUBA4A; TUBB1	Both
Melphalan	1992	Multiple myeloma; Ovarian cancer	DNA synthesis	Combination
Teniposide	1992	Leukemia	TOP2A	Combination
Cladribine	1993	Leukemia	DNA synthesis	Single
Vinorelbine tartrate	1994	Lung cancer	TUBB	Both
Pegaspargase	1994	Leukemia	Biological	Combination
Thiotepa	1994	Breast cancer; Ovarian cancer; Bladder cancer	DNA synthesis	Single
Docetaxel	1996	Prostate cancer; Breast cancer; Head and neck cancer; Stomach cancer; Lung cancer; Brain cancer	TUBA4A; TUBB1	Both
Gemcitabine	1996	Ovarian cancer; Pancreatic cancer; Lung cancer; Breast cancer	DNA synthesis; RRM1; TYMS	Both
Irinotecan	1996	Colorectal cancer	TOP1; TOP1MT	Both
Topotecan hydrochloride	1996	Ovarian cancer; Lung cancer; Cervical cancer	TOP1; TOP1MT	Both
Idarubicin	1997	Leukemia	DNA synthesis; TOP2A	Combination
Capecitabine	1998	Colorectal cancer; Breast cancer	DNA synthesis; RNA synthesis; Protein synthesis; TYMS	Both
Daunorubicin hydrochloride	1998	Leukemia	DNA synthesis; TOP2A; TOP2B	Combination
Valrubicin	1998	Bladder cancer	DNA synthesis; TOP2A	Single
Temozolomide	1999	Brain cancer	DNA synthesis	Both
Cytarabine	1999	Leukemia	DNA synthesis	Single
Epirubicin	1999	Breast cancer	CHD1; DNA synthesis; TOP2A	Single
Arsenic trioxide	2000	Leukemia	Unknown	Single
Mitomycin	2002	Stomach cancer; Pancreatic cancer	DNA synthesis	Both
Oxaliplatin	2002	Colorectal cancer	DNA synthesis	Combination
Pemetrexed disodium	2004	Lung cancer; Mesothelioma	DHFR; GART; TYMS	Both
Clofarabine	2004	Leukemia	DNA synthesis	Single
Nelarabine	2005	Leukemia; Lymphoma	DNA synthesis	Single
Ixabepilone	2007	Breast cancer	TUBB3	Both
Bendamustine hydrochloride	2008	Leukemia; Lymphoma	DNA synthesis	Single
Pralatrexate	2009	Lymphoma	DHFR; TYMS	Single
Cabazitaxel	2010	Prostate cancer	TUBA4A; TUBB1	Combination
Eribulin mesylate	2010	Breast cancer	TUBA4A; TUBB1	Single
Asparaginase erwinia chrysanthemi	2011	Leukemia	Biological	Combination
Omacetaxine mepesuccinate	2012	Leukemia	RPL3	Single
Radium 223 dichloride	2013	Prostate cancer	Unknown	Single
Targeted				
Fluoxymesterone	1956	Breast cancer	AR; ESR1; NR3C1; PRLR	Single
Methyltestosterone	1973	Breast cancer	AR	Single
Tamoxifen citrate	1977	Breast cancer	ESR1; ESR2	Single
Estramustine	1981	Prostate cancer	ESR1; ESR2; MAP1A; MAP2	Single
Interferon Alfa-2b, recombinant	1986	Sarcoma; Leukemia; Melanoma; Lymphoma	IFNAR1; IFNAR2	Single
Goserelin	1989	Prostate cancer; Breast cancer	GNRHR; LHCGR	Both
Flutamide	1989	Prostate cancer	AR	Combination
Aldesleukin	1992	Melanoma; Kidney cancer	IL2RA; IL2RB; IL2RG	Single
Bicalutamide	1995	Prostate cancer	AR	Combination
Anastrozole	1995	Breast cancer	CYP19A1	Single
Porfimer	1995	Esophageal cancer; Lung cancer	FCGR1A; LDLR	Single
Nilutamide	1996	Prostate cancer	AR	Combination
Imiquimod	1997	Basal cell carcinoma	TLR7; TLR8	Single
Letrozole	1997	Breast cancer	CYP19A1	Single
Rituximab	1997	Lymphoma; Leukemia	MS4A1	Single
Toremifene	1997	Breast cancer	ESR1	Single
Thalidomide	1998	Multiple myeloma	CRBN	Combination
Trastuzumab	1998	Breast cancer; Stomach cancer	ERBB2	Single
Alitretinoin	1999	Kaposi’s sarcoma	RARA; RARB; RARG; RXRA; RXRB; RXRG	Single
Bexarotene	1999	Lymphoma	RXRA; RXRB; RXRG	Single
Denileukin diftitox	1999	Lymphoma	IL2RA; IL2RB; IL2RG; protein synthesis	Single
Exemestane	1999	Breast cancer	CYP19A1	Single
Gemtuzumab ozogamicin	2000	Leukemia	CD33; DNA synthesis	Single
Triptorelin	2000	Prostate cancer	GNRH1	Single
Alemtuzumab	2001	Leukemia	CD52	Single
Imatinib mesylate	2001	Leukemia; Stomach cancer	BCR-ABL	Single
Peginterferon Alfa-2b	2001	Melanoma	IFNAR1; IFNAR2	Single
Fulvestrant	2002	Breast cancer	ESR1	Single
Ibritumomab tiuxetan	2002	Lymphoma	MS4A1	Single
Leuprolide acetate	2002	Prostate cancer	GNRHR	Single
Abarelix	2003	Prostate cancer	GNRHR	Single
Bortezomib	2003	Multiple myeloma; Lymphoma	PSMB1; PSMB2; PSMB5; PSMD1; PSMD2	Single
Gefitinib	2003	Lung cancer	EGFR	Single
Tositumomab and Iodine I 131 Tositumomab	2003	Lymphoma	MS4A1	Single
Bevacizumab	2004	Colorectal cancer; Lung cancer; Brain cancer; Kidney cancer	VEGFA	Both
Cetuximab	2004	Head and neck cancer; Colorectal cancer	EGFR	Both
Erlotinib hydrochloride	2004	Pancreatic cancer; Lung cancer	EGFR	Both
Azacitidine	2004	Leukemia	DNMT1	Single
Lenalidomide	2005	Multiple myeloma; Lymphoma	CRBN	Both
Sorafenib tosylate	2005	Liver cancer; Kidney cancer; Thyroid cancer	BRAF; FGFR1; FLT1; FLT3; FLT4; KDR; KIT; PDGFRB; RAF1; RET	Single
Dasatinib	2006	Leukemia	BCR-ABL	Single
Decitabine	2006	Leukemia	DNMT1	Single
Panitumumab	2006	Colorectal cancer	EGFR	Single
Sunitinib malate	2006	Stomach cancer; Kidney cancer; Pancreatic cancer	CSF1R; FLT1; FLT3; FLT4; KDR; KIT; PDGFRA; PDGFRB	Single
Vorinostat	2006	Lymphoma	HDAC1; HDAC2; HDAC3; HDAC6	Single
Lapatinib ditosylate	2007	Breast cancer	EGFR; ERBB2	Combination
Nilotinib	2007	Leukemia	BCR-ABL	Single
Temsirolimus	2007	Kidney cancer	MTOR	Single
Degarelix	2008	Prostate cancer	GNRHR	Single
Everolimus	2009	Breast cancer; Brain cancer; Kidney cancer; Pancreatic cancer	MTOR	Both
Ofatumumab	2009	Leukemia	MS4A1	Single
Pazopanib hydrochloride	2009	Kidney cancer; Sarcoma	FGF1; FGFR3; FLT1; FLT4; ITK; KDR; KIT; PDGFRA; PDGFRB; SH2B3	Single
Romidepsin	2009	Lymphoma	HDAC1; HDAC2; HDAC3; HDAC6	Single
Denosumab	2010	Bone cancer	TNFSF11	Single
Hydroxyurea	2010	Melanoma; Leukemia; Ovarian cancer; Head and neck cancer	RRM1	Single
Sipuleucel-T	2010	Prostate cancer	ACPP	Single
Abiraterone acetate	2011	Prostate cancer	CYP17A1	Single
Brentuximab vedotin	2011	Lymphoma	TNFRSF8	Single
Crizotinib	2011	Lung cancer	ALK; MET	Single
Ipilimumab	2011	Melanoma	CTLA4	Single
Ruxolitinib phosphate	2011	Myelofibrosis	JAK1; JAK2	Single
Vandetanib	2011	Thyroid cancer	EGFR; PTK6; TEK; VEGFA	Single
Vemurafenib	2011	Melanoma	BRAF	Single
Pertuzumab	2012	Breast cancer	ERBB2	Both
Axitinib	2012	Kidney cancer	FLT1; FLT4; KDR	Single
Bosutinib	2012	Leukemia	BCR-ABL	Single
Cabozantinib	2012	Thyroid cancer	KDR; MET; RET	Single
Carfilzomib	2012	Multiple myeloma	PSMB1; PSMB10; PSMB2; PSMB5; PSMB8; PSMB9	Single
Enzalutamide	2012	Prostate cancer	AR	Single
Ponatinib hydrochloride	2012	Leukemia	BCR-ABL	Single
Regorafenib	2012	Colorectal cancer; Stomach cancer	RET; FLT1; KDR; FLT4; KIT; PDGFRA; PDGFRB; FGFR1; FGFR2; TEK; DDR2; NTRK1; EPHA2; RAF1; BRAF; MAPK11; FRK; ABL1	Single
Vismodegib	2012	Basal cell carcinoma	SMO	Single
Ziv-aflibercept	2012	Colorectal cancer	PGF; VEGFA; VEGFB	Single
Dabrafenib	2013	Melanoma	BRAF; LIMK1; NEK11; RAF1; SIK1	Both
Trametinib	2013	Melanoma	MAP2K1; MAP2K2	Both
Obinutuzumab	2013	Leukemia	MS4A1	Combination
Ado-trastuzumab emtansine	2013	Breast cancer	ERBB2	Single
Afatinib	2013	Lung cancer	EGFR; ERBB2; ERBB4	Single
Ibrutinib	2013	Lymphoma	BTK	Single
Pomalidomide	2013	Multiple myeloma	CRBN	Single
Idelalisib	2014	Leukemia; Lymphoma	PIK3CD	Both
Belinostat	2014	Lymphoma	HDAC1; HDAC2; HDAC3; HDAC6	Single
Ceritinib	2014	Lung cancer	ALK	Single
Pembrolizumab	2014	Melanoma	PDCD1	Single
Ramucirumab	2014	Stomach cancer	KDR	Single
Lanreotide	2014	Gastroenteropancreatic neuroendocrine tumor	SSTR2; SSTR5	Single
Blinatumomab	2014	Leukemia	CD19; CD3D	Single
Nivolumab	2014	Melanoma	PDCD1	Single
Olaparib	2014	Ovarian cancer	PARP1; PARP2; PARP3	Single

Among 89 targeted drugs, 18 are antibodies, of which two (rituximab and trastuzumab) were approved in 1990, eight in the 2000s (gemtuzumab ozogamicin, alemtuzumab, ibritumomab tiuxetan, tositumomab and iodine I 131 tositumomab, bevacizumab, cetuximab, panitumumab, and ofatumumab) and seven from 2010 to 2014 (denosumab, brentuximab vedotin, ipilimumab, pertuzumab, ado-trastuzumab emtansine, obinutuzumab, and pembrolizumab). The trend was consistent with previous observations [7], which indicated that the advanced molecular understanding of cancer during the period had contributed substantially to the development of the anticancer drug, especially targeted drugs [30].

**Fig. 2:**
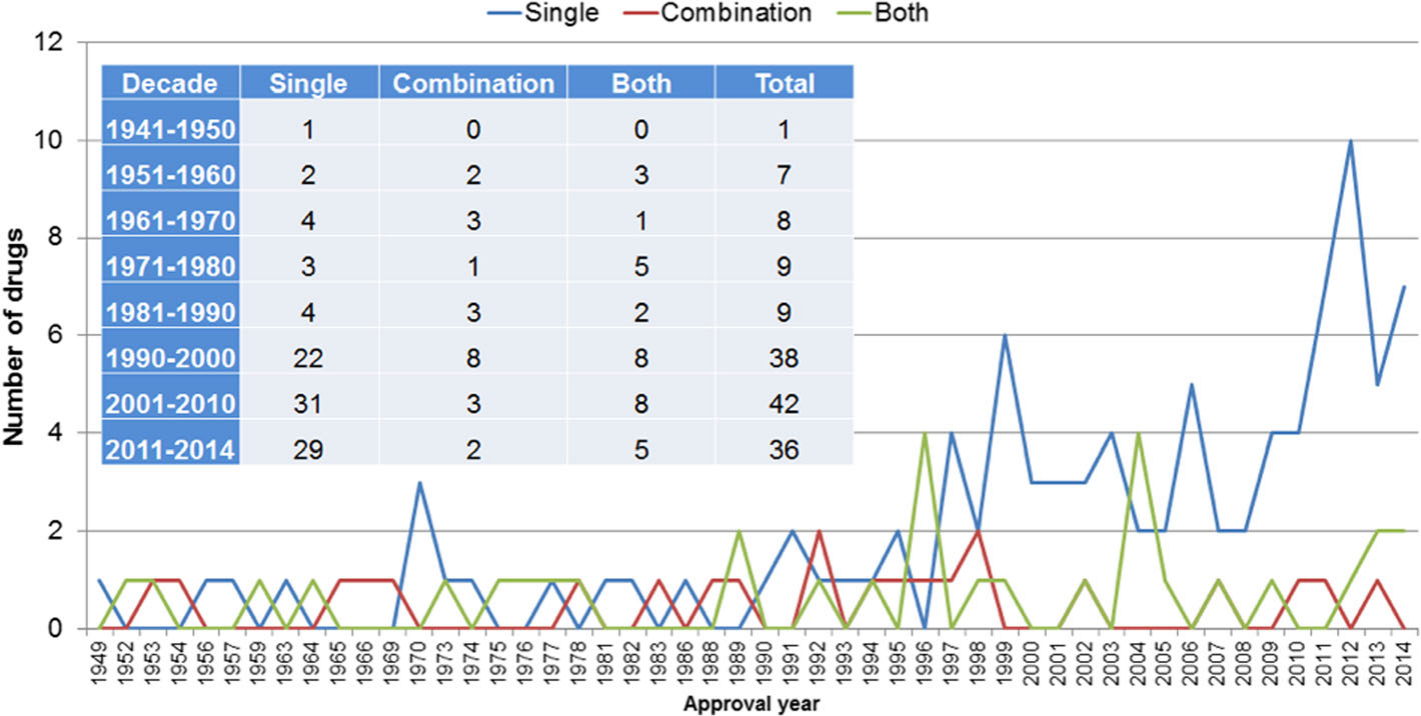
Delivery methods of anticancer drugs approved by FDA from 1949 to 2014

According to the drug delivery method administered to the patient, one drug can be categorized as a cancer single (individual) drug or a cancer combination drug. A combination drug is a drug that makes up a cancer drug combination that several individual drugs are administered to the patient. Though the targeted agents have become the primary focus of the therapeutic cancer research, investigation of their combined use with other targeted drugs or with cytotoxic drugs has become promising for the development of the effective cancer treatment [31, 32]. Among the 150 drugs, 96 drugs could be given to patients one at a time, 22 could be given in combination with other cancer drugs to patients, and 32 drugs could be delivered to patients as the combination drugs or single drugs (Fig. 2). The targeted drugs tended to be delivered as signal drugs (Pearson’s correlation: *r* = 0.92, *P* < 2.2 × 10^−26^) while cytotoxic drug tended to be delivered as combination drugs (*r* = 0.43, *P* = 0.002) or by both methods (*r* = 0.44, *P* = 0.001).

### Subcellular location and function of drug targets

In our curated data set, among the 150 anticancer FDA-approved drugs, 89 were targeted drugs that could be used to treat 23 types of cancer and acted on 102 protein targets (Tables 1, 2). To comprehensively understand the target functions and their genetic roles in cancer, we performed a survey from the perspectives of subcellular location, functional classification, and genetic mutations. These insights might be valuable for further understanding of molecular mechanisms of cancer and the advanced development of cancer therapy [30, 33, 34].

**Table 2:** Subcellular location and function classification of targeted drug targets

Subcellular location	Family	Subfamily	Number of targets
Cytoplasm (27)			
	Enzyme (23)	E3 ligase	1
		Epigenetic enzyme	1
		Monooxygenases	2
		Peptidase	6
		Phosphatidyl Inositol Kinases	1
		Serine/threonine kinase	5
		Threonine/tyrosine-protein kinase	2
		Tyrosine kinase	5
	Other (4)	Other	4
Extracellular space (7)			
	Cytokine (1)	Cytokine	1
	Enzyme (1)	Phosphatase	1
	Growth factor (4)	Growth factor	4
	Hormone (1)	Hormone	1
Nucleus (23)			
	Enzyme (13)	Epigenetic enzyme	4
		Polymerase	3
		Ribonucleotide diphosphate reductase	1
		Serine/threonine kinase	3
		Tyrosine kinase	2
	Receptor(10)	Ligand-dependent nuclear receptor	10
Plasma membrane (45)			
	Antigen (5)	Antigen	5
	Enzyme(21)	Tyrosine kinase	21
	Receptor(17)	Transmembrane receptor	12
		G-protein coupled receptor	5
	Transporter (1)	Transporter	1
	Other (1)	Other	1

**Fig. 3:**
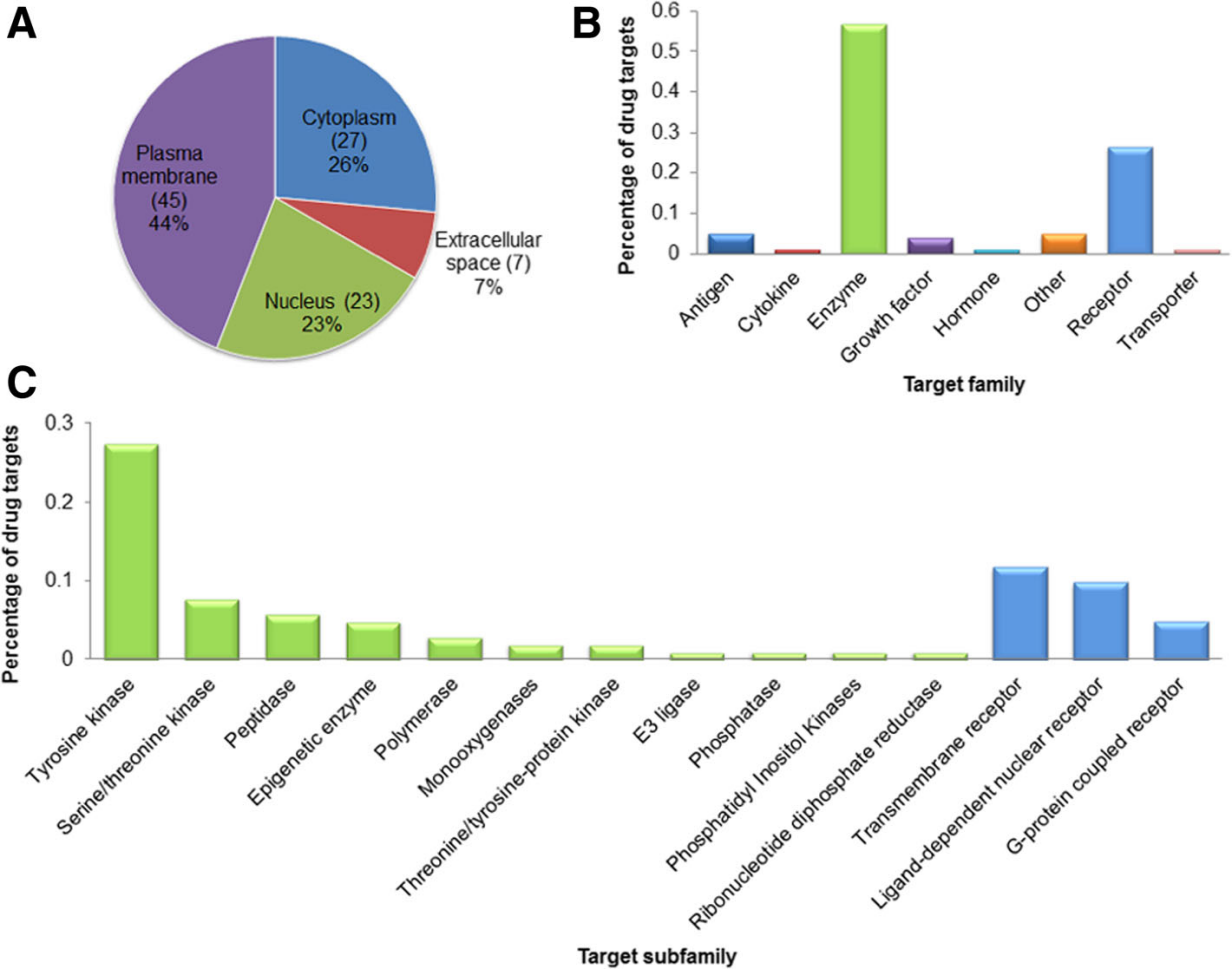
Anticancer drug target percentage of subcellular locations **a** and function families **b** and **c**

We retrieved the target’s subcellular information and function classification from IPA and manually reviewed for each target (Table 2). The result shows that most of the drug targets (45, 44%) located in the plasma membrane, 27 (26%) in the cytoplasm, 23 (23%) in the cell nucleus, and only seven (7%) in the extracellular space (Fig. 3a). Among the 45 targets in the plasma membrane, 21 were tyrosine kinases, 12 were transmembrane receptors, five were antigens, and five were G-protein coupled receptors. Among the 27 targets in the cytoplasm, 23 were enzymes and four were others. Among the 23 targets in the nucleus, 13 were enzymes and 10 were receptors. The observation indicates that, to date, the most successful anticancer drugs target the plasma membrane proteins.

The data set showed that enzymes made up the largest groups of drug targets (58, 57%) while receptors were the second largest group of anticancer target proteins (27, 26%) (Fig. 3b). Of these enzymes, 28 (27%) were tyrosine kinases, eight (8%) were the serine/threonine kinases, six (6%) were peptidases, and five (5%) were epigenetic enzymes (Fig. 3c). Of these receptors, 12 (12%) were transmembrane receptors, 10 (10%) were ligand-dependent nuclear receptors, and five (5%) were G-protein coupled receptors (Fig. 3c).

### Genetic pattern of targeted anticancer targets

To check if these targets are the cancer candidate genes, we compared them with the cancer gene set which contains 594 genes from the Cancer Gene Census [26]. Among 102 target genes, 32 genes are cancer genes. Compared to all the protein-coding genes in the human (20,729), the anticancer drug targets were significantly enriched with cancer genes (Hypergeometric test, *P*-value = 3.57 × 10^−25^). Among the 32 cancer genes, 16 were oncogenes while none were tumor suppressor genes according to the the high confidence TSGs and OCGs from Davioli et al. [27].

**Fig. 4:**
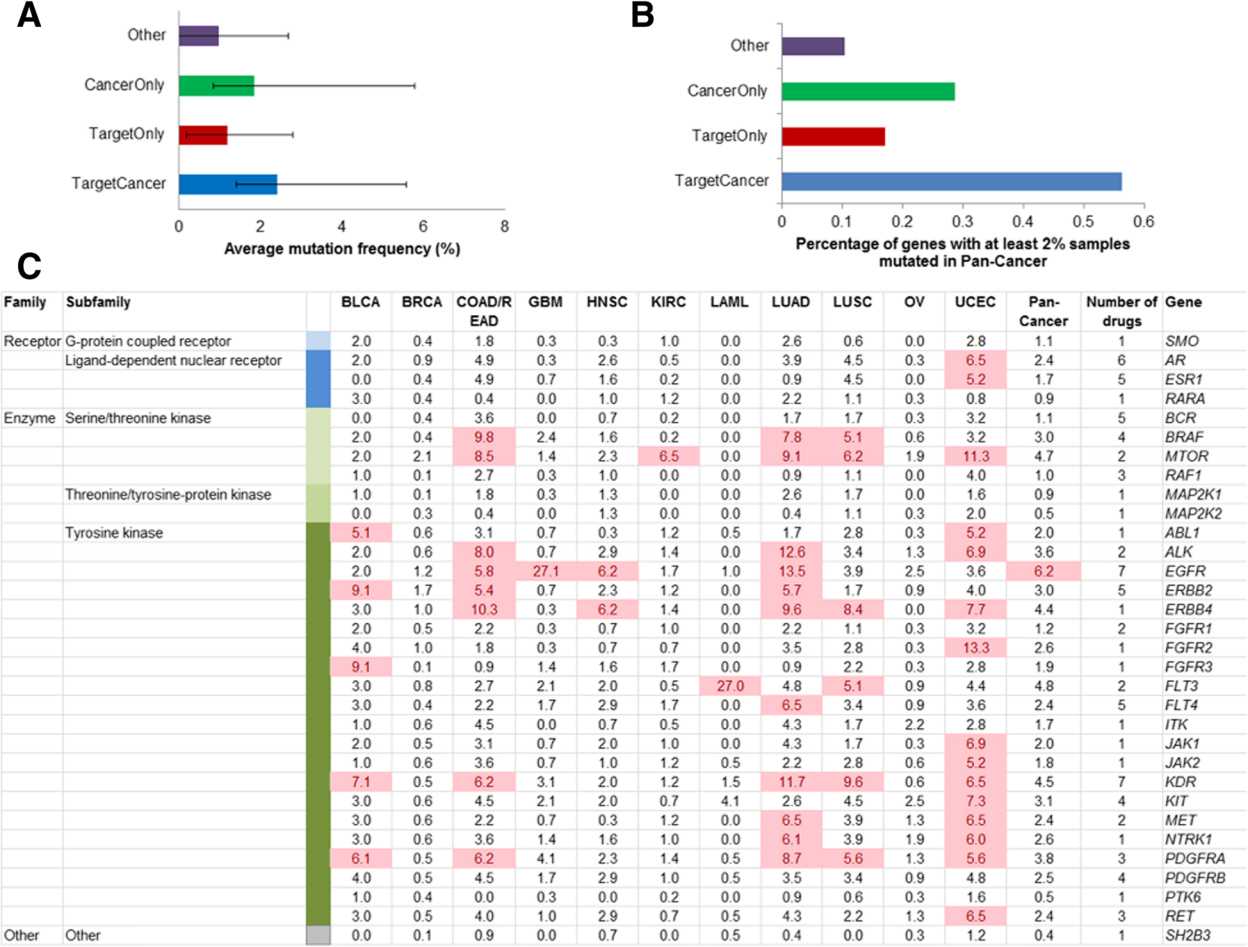
Mutation pattern of drug target genes belonging to cancer genes. The TargetCancer represented the common genes between anticancer drug targets and cancer genes. The TargetOnly represented the genes only belonging to genes encoding drug targets with mutation data. The CancerOnly represented the genes only belonging to cancer genes with mutation data. The Other represented genes with mutation data excluding the genes from above three gene sets. **a** Comparison of average mutation frequency of four gene sets. **b** Percentage of genes with at least 2% mutation frequency in the Pan-Cancer. **c** The function classification, mutation frequency in individual cancer type and Pan-Cancer, and numbers of drugs of 32 TargetCancer genes. We highlighted the mutation frequency higher than 5% of samples in “TargetCancer” genes with *red color*

To further explore the mutation pattern of the anticancer drug targets, we utilized the somatic mutations in 3268 patients across 12 types of cancer from TCGA Pan-Cancer [28]. Among 102 drug targets, 32 were cancer genes. Thus we compared the mutation frequency of four gene sets: 32 genes belonging to drug targets and cancer genes (TargetCancer genes), 70 genes only belonging to genes encoding drug targets (TargetOnly genes), 537 cancer genes only belonging to cancer genes and with mutation data (CancerOnly genes), and 20,308 genes with mutation data excluding the genes from above three gene sets (Other genes). To compare the distribution of mutation frequency of the tumor samples among the four gene sets, we performed the Kolmogorov-Smirnor (K-S) tests. Figure 4a shows the comparison of mutation percentage of all samples in each gene set. The TargetCancer genes had the highest average mutation frequency (2.41%), which was significantly higher than that of TargetOnly (1.19%, K-S test: *P* = 4.79 × 10^−5^), CancerOnly (1.85%, *P* = 0.0005), and Other genes (0.97%, *P* = 1.31 × 10^−9^). The CancerOnly genes had the second highest average mutation frequency (1.85%), which was significantly higher than that of of TargetOnly (*P* = 0.0275) and other genes (*P* < 2.2× 10^−17^). The TargetOnly genes had the third highest average mutation frequency, which was significantly higher than that of other genes (*P* = 0.0134).

Notably, among the 32 TargetCancer genes, 18 genes (56%) had at least 2% mutation frequency across the Pan-Cancer collection (Fig. 4b). Compared to that of TargetOnly genes (39%), CancerOnly (29%), or Other gene sets (10%), the percentage was significantly higher (Chi-squared test *P*-values: 0.0002, 0.002, 2.48 × 10^−16^, respectively). Figure 4c shows the percentage of samples with mutations of the 32 TargetCancer genes, their function classification, and number of targeting drugs. Indeed, for the 32 Target Cancer genes, there was a significant correlation between the percentages of samples with mutations and numbers of targeted drugs (Pearson’s correlation: *r* = 0.40, *P* = 0.0230). Among the 32 genes, the most frequently mutated gene in the Pan-Cancer cohort was *EGFR* (6.2%). Its mutations significantly occur in the brain cancer GBM (27.1%), lung cancer (13.5%), COAD/READ (5.8%), HNSC (6.2%). Among the seven drugs targeting the gene, three (afatinib, erlotinib, and gefitinib) were used to treat lung cancer, two (cetuximab and panitumumab) were used to treat colorectal cancer, and one (cetuximab) was used to treat head and neck cancer.

### Drug-cancer network

**Fig. 5:**
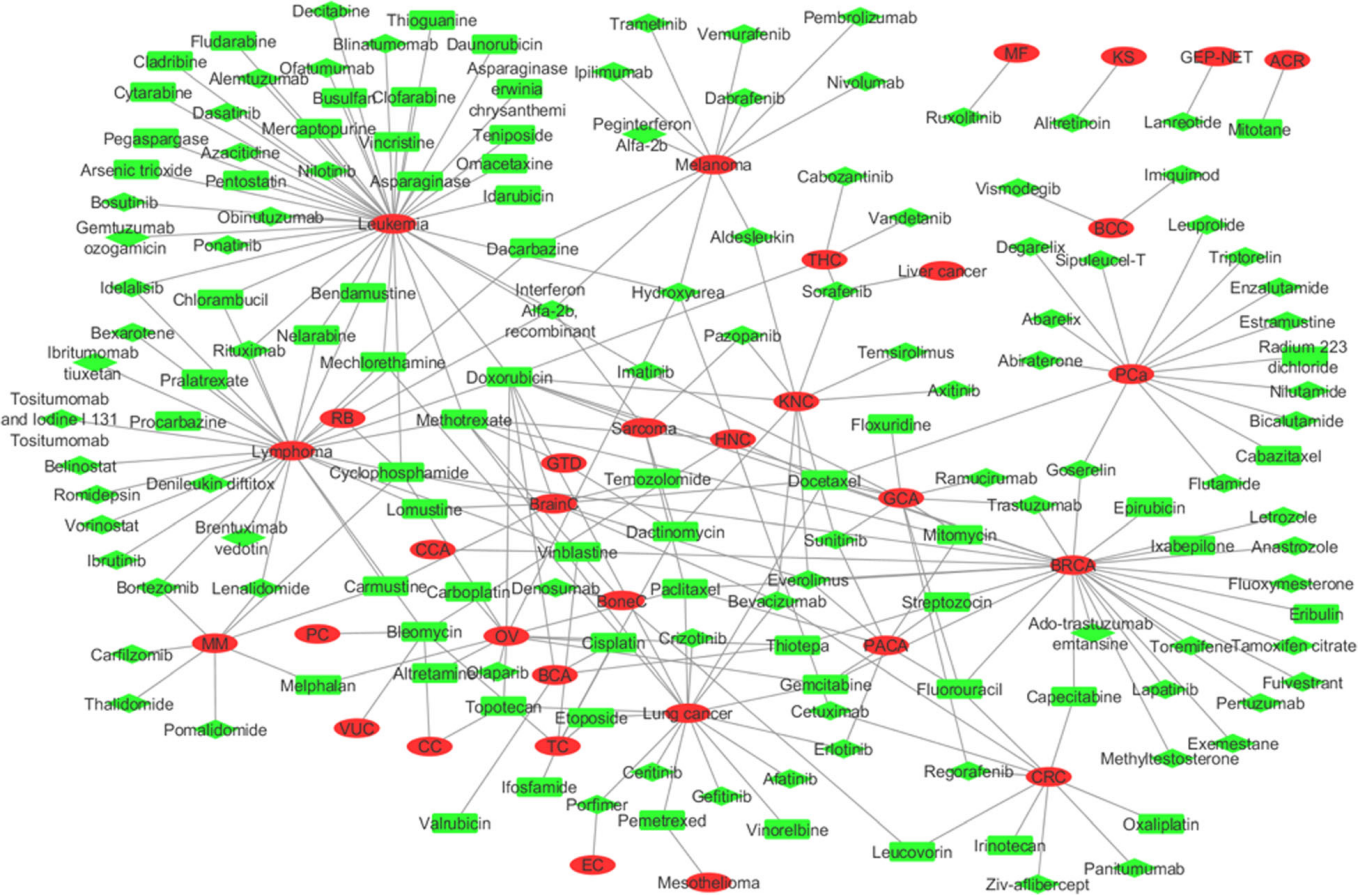
Drug-cancer network. The *red ellipse* represents the cancer; the *green rectangle* represents the cytotoxic drug; the *green diamond* represents the targeted drug. The cancer abbreviations included in the Table 3

To explore the associations between the drugs and cancer types, we generated a drug-cancer network, which comprised 183 nodes (150 drugs and 33 cancer types) and 248 drug-cancer associations (Fig. 5) based on the FDA-approved drug-cancer associations in our curated data.

In the drug-cancer network, the degree (number of cancer types) of the 150 drugs ranged from one to eleven, and the average degree was 1.65. The degree distribution of these drugs was strongly right-skewed, indicating that most drugs had a low degree and only a small portion of the nodes had a high degree. The degree of the cytotoxic drugs was 2.13, which was significantly higher than that of the targeted drugs (1.33, K-S test: *P* = 0.0378). Most of them (105, 70%) could be used to treat only one cancer type. Among the 105 drugs, 35 belonged to the cytotoxic drugs while 70 belonged to the targeted drugs. Among the rest 45 drugs, 24 (16%) could be used to treat two cancer types and 21 drugs (14%) could be used to treat at least three cancer types. Among the 21 drugs, 15 were cytotoxic drugs while six were targeted drugs. Most of the 21 drugs (16, 76%) were approved by FDA before 2000. The most commonly used drug was doxorubicin that could be used to treat 11 cancer types, including leukemia, breast cancer, stomach cancer, lymphoma, ovarian cancer, lung cancer, sarcoma, thyroid cancer, bladder cancer, kidney cancer, and brain cancer. Doxorubicin is a cytotoxic anthracycline antibiotic isolated from cultures of Streptomyces peucetius var. caesius, which binds to nucleic acids, presumably by specific intercalation of the planar anthracycline nucleus with the DNA double helix [35]. The result indicated that the cytotoxic drugs tended to be used to treat more cancer types than targeted drugs.

In the drug-cancer network, the degree (number of drugs) of the 33 cancer types ranged from one to 40 and the average degree was 7.52. The degree distribution of the cancer types was not obviously right-skewed. Among the 33 cancer types, 11 had one drug, 12 had at least two drugs and less than 10 drugs, and ten had at least ten drugs (Table 3). They were leukemia (number of drugs: 40), lymphoma (28), breast cancer (27), lung cancer (17), prostate cancer (15), ovarian cancer (12), melanoma (11), colorectal cancer (10), kidney cancer (10), and stomach cancer (10). Among the 40 drugs used to treat leukemia, 24 belonged to cytotoxic drugs while 16 drugs were the targeted drugs. Similarly, the numbers of cytotoxic drugs and targeted drugs were similar to each other for lymphoma, breast cancer, and lung cancer. However, for prostate cancer, melanoma, and kidney cancer, the numbers of targeted drugs were significantly higher than those of cytotoxic drugs.

**Table 3:** Cancer classes, their abbreviations, and number of anticancer drugs

Cancer	Abbreviation	Number of drugs	Number of targeted drugs	Number of cytotoxic drugs
Leukemia	Leukemia	40	16	24
Lymphoma	Lymphoma	28	14	14
Breast cancer	BRCA	27	14	13
Lung cancer	Lung cancer	17	7	10
Prostate cancer	PCa	15	12	3
Ovarian cancer	OV	12	2	10
Melanoma	Melanoma	11	10	1
Colorectal cancer	CRC	10	5	5
Kidney cancer	KNC	10	8	2
Stomach cancer	GCA	10	5	5
Brain cancer	BrainC	8	2	6
Multiple myeloma	MM	8	5	3
Pancreatic cancer	PACA	8	3	5
Testicular cancer	TC	6	0	6
Head and neck cancer	HNC	5	2	3
Sarcoma	Sarcoma	5	2	3
Bladder cancer	BCA	4	0	4
Thyroid cancer	THC	4	3	1
Bone cancer	BoneC	3	1	2
Basal cell carcinoma	BCC	2	2	0
Cervical cancer	CC	2	0	2
Gestational trophoblastic disease	GTD	2	0	2
Adrenal cortical carcinoma	ACR	1	0	1
Choriocarcinoma	CCA	1	0	1
Esophageal cancer	EC	1	1	0
Gastroenteropancreatic neuroendocrine tumor	GEP-NET	1	1	0
Kaposi’s sarcoma	KS	1	1	0
Liver cancer	Liver cancer	1	1	0
Mesothelioma	Mesothelioma	1	0	1
Myelofibrosis	MF	1	1	0
Penile cancer	PC	1	0	1
Retinoblastoma	RB	1	0	1
Vulvar cancer	VUC	1	0	1

### Network of targeted drugs, targets, and cancer

**Fig. 6:**
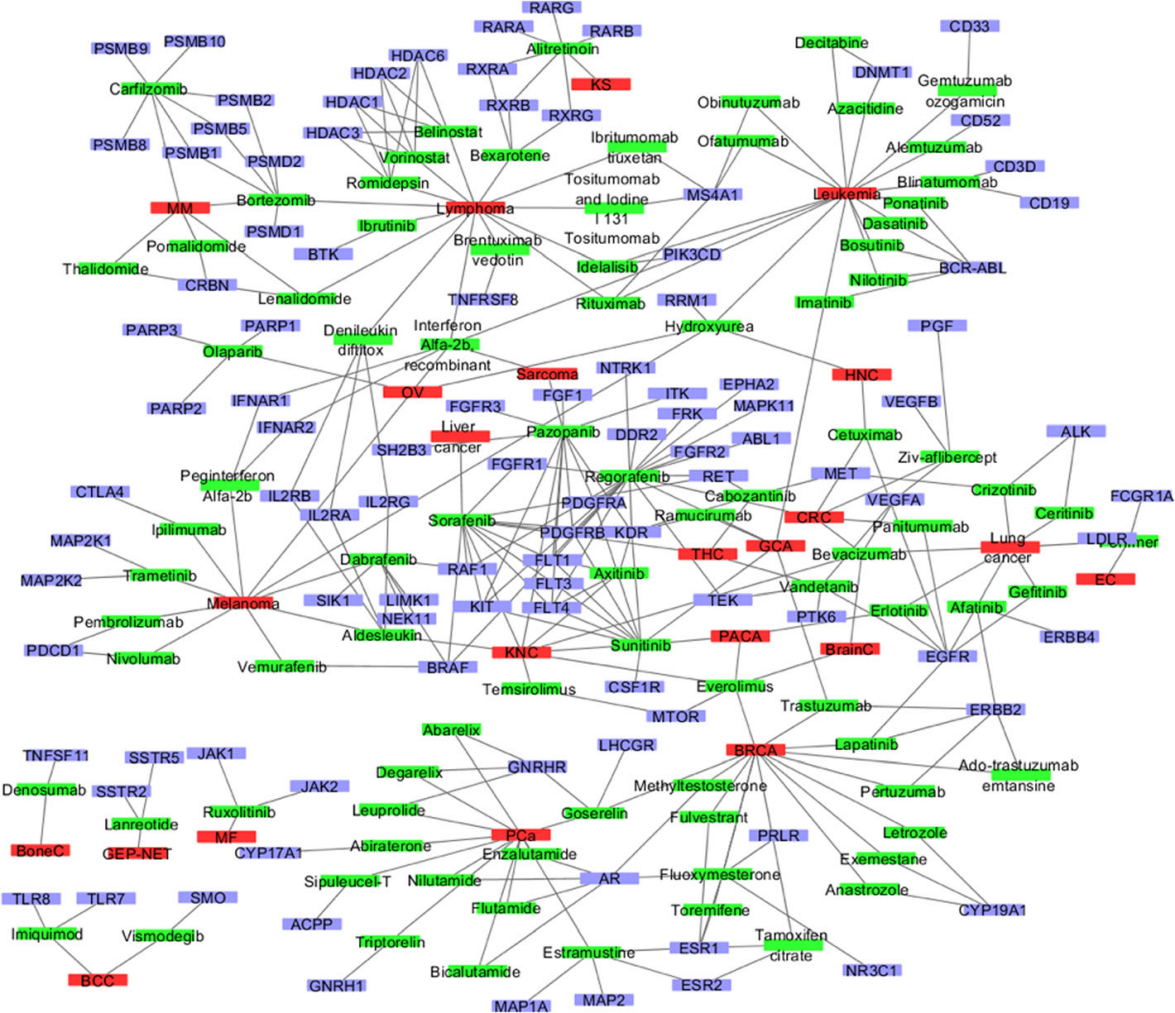
Network of targeted drugs, targets, and cancer types. The *red rectangle* represents the cancer; the *green rectangle* represents the targeted drug, the *blue rectangle* represents the drug target. The cancer abbreviations included in the Table 3

Besides the drug-cancer network, we generated a specific network for targeted drugs, their targets, and their indications. The network contained 214 nodes (89 drugs, 102 targets, and 23 cancer types) and 313 edges (118 drug-cancer associations and 195 drug-target associations) (Fig. 6) based on the FDA-approved targeted drug-cancer associations and targeted drug-target associations in our curated data.

In the network, drugs had two types of neighbors: drug target and drug indication (cancer type). The target degree (number of targets) of the 89 drugs ranged from one to 18, and the average degree was 2.19. The cancer degree (number of cancer types) of the 89 drugs ranged from one to four and the average degree was 1.33. Among the 89 drugs, 22 had more than two targets. The drug regorafenib had 18 targets, which was approved by FDA to treat gastrointestinal stromal tumors and metastatic colorectal cancer. Among the 89 drugs, 19 drugs could be used to treat more than one cancer types. Four drugs bevacizumab, everolimus, hydroxyurea, and recombinant interferon Alfa-2b could be used to treat four types of cancer. The degree (number of drugs) of targets ranged from one to seven and the average degree was 1.91. The EGFR (epidermal growth factor receptor) and KDR (kinase insert domain receptor) were the most popular targets and both could be targeted by seven drugs, separately. The EGFR-related seven drugs could be used to treat six cancer types, while KDR-related drugs could be used to treat seven types of cancer. There were three common cancer types: colorectal cancer, thyroid cancer, pancreatic cancer. The degree (number of drugs) of cancer types ranged from one to 16 and the average degree was 5.13. As we discussed before, leukemia had 16 targeted drugs can be used to treat.

The common target-based approach, namely, the drugs that shared common targets could be used to treat the same disease, is one of the “guilt-by-association” strategies to identify the novel drug-disease associations [29]. During the analysis, we noticed that, among the 89 drugs, 70 drugs had at least one common target. Applying the common target-based approach, we discovered 133 novel drug-cancer associations among 52 drugs and 16 cancer types. To evaluate the novel drug-cancer associations, we utilized the clinical trial studies to see if the drug had been investigated in the corresponding cancer type. After searching using the 52 drugs and their predicted cancer types against ClinivalTrials.gov, we found that most of the drug-cancer associations (116) have been investigated in at least one clinical trial (Table 4) while the 17 had not been investigated in clinical trials. The later part of novel drug-cancer associations might provide valuable clues for drug repurposing. The most well-studied association was the thalidomide-lymphoma, which had 174 clinical trial studies, including 15 Phase III clinical trial studies and one Phase IV clinical trial study. The drug thalidomide was approved to treat multiple myeloma. Recently its combination with other drugs entered to treat the peripheral T-cell lymphoma in the Phase 4 study (ClinicalTrials.gov Identifier: NCT01664975).

**Table 4:** Potential drug-cancer associations with numbers of clinical trials

Drug	Possible indication	Number of clinical trials^a^	Drug	Possible indication	Number of clinical trials^a^
Thalidomide	Lymphoma	174	Ziv-aflibercept	Lung cancer	5
Temsirolimus	BRCA	129	Afatinib	CRC	4
Cetuximab	Lung cancer	77	Axitinib	THC	4
Ofatumumab	Lymphoma	64	Gefitinib	PACA	4
Erlotinib	HNC	62	Pazopanib	GCA	4
Temsirolimus	PACA	50	Pazopanib	CRC	4
Aldesleukin	Lymphoma	44	Pertuzumab	GCA	4
Obinutuzumab	Lymphoma	44	Regorafenib	KNC	4
Gefitinib	HNC	43	Regorafenib	PACA	4
Temsirolimus	BrainC	40	Regorafenib	Melanoma	4
Axitinib	KNC	39	Tamoxifen citrate	PCa	4
Cetuximab	PACA	31	Tositumomab and Iodine I 131 Tositumomab	Leukemia	4
Erlotinib	CRC	31	Vandetanib	KNC	4
Panitumumab	HNC	31	Vemurafenib	THC	4
Sorafenib	Melanoma	30	Ziv-aflibercept	KNC	4
Vandetanib	Lung cancer	29	Ado-trastuzumab emtansine	GCA	3
Erlotinib	BRCA	25	Afatinib	PACA	3
Sorafenib	PACA	25	Bevacizumab	THC	3
Sorafenib	CRC	23	Cabozantinib	PACA	3
Trastuzumab	Lung cancer	22	Cabozantinib	Sarcoma	3
Vandetanib	HNC	22	Dabrafenib	THC	3
Carfilzomib	Lymphoma	20	Fulvestrant	PCa	3
Lapatinib	HNC	20	Pertuzumab	Lung cancer	3
Afatinib	HNC	17	Vandetanib	PACA	3
Sunitinib	Sarcoma	17	Ziv-aflibercept	BrainC	3
Panitumumab	Lung cancer	16	Axitinib	Sarcoma	2
Sorafenib	Sarcoma	16	Axitinib	PACA	2
Sunitinib	Liver cancer	16	Axitinib	GCA	2
Cetuximab	BRCA	14	Cabozantinib	Liver cancer	2
Peginterferon Alfa-2b	Leukemia	14	Denileukin diftitox	KNC	2
Sunitinib	CRC	14	Estramustine	BRCA	2
Lapatinib	GCA	13	Gefitinib	THC	2
Gefitinib	CRC	12	Vandetanib	GCA	2
Gefitinib	BRCA	12	Bexarotene	KS	1
Leuprolide	BRCA	12	Cabozantinib	CRC	1
Vandetanib	BRCA	11	Cabozantinib	GCA	1
Sorafenib	GCA	10	Cetuximab	THC	1
Bicalutamide	BRCA	9	Crizotinib	THC	1
Denileukin diftitox	Melanoma	9	Dabrafenib	KNC	1
Enzalutamide	BRCA	9	Dabrafenib	Liver cancer	1
Ibritumomab tiuxetan	Leukemia	9	Dabrafenib	CRC	1
Cabozantinib	Lung cancer	8	Degarelix	BRCA	1
Regorafenib	Liver cancer	8	Erlotinib	THC	1
Lapatinib	Lung cancer	7	Lapatinib	THC	1
Lapatinib	CRC	7	Peginterferon Alfa-2b	Sarcoma	1
Panitumumab	PACA	7	Ramucirumab	PACA	1
Ramucirumab	Liver cancer	7	Ramucirumab	Sarcoma	1
Vandetanib	CRC	7	Regorafenib	THC	1
Vandetanib	BrainC	7	Ziv-aflibercept	THC	1
Ado-trastuzumab emtansine	Lung cancer	6	Abarelix	BRCA	0
Axitinib	Liver cancer	6	Afatinib	THC	0
Cabozantinib	KNC	6	Alitretinoin	Lymphoma	0
Pazopanib	PACA	6	Bosutinib	GCA	0
Pazopanib	THC	6	Dabrafenib	GCA	0
Ramucirumab	CRC	6	Dasatinib	GCA	0
Sunitinib	THC	6	Fluoxymesterone	PCa	0
Afatinib	GCA	5	Flutamide	BRCA	0
Axitinib	CRC	5	Methyltestosterone	PCa	0
Lapatinib	PACA	5	Nilotinib	GCA	0
Panitumumab	BRCA	5	Nilutamide	BRCA	0
Pazopanib	Liver cancer	5	Panitumumab	THC	0
Peginterferon Alfa-2b	Lymphoma	5	Ponatinib	GCA	0
Pomalidomide	Lymphoma	5	Ramucirumab	THC	0
Ramucirumab	KNC	5	Vemurafenib	GCA	0
Regorafenib	Sarcoma	5	Vemurafenib	KNC	0
Toremifene	PCa	5	Vemurafenib	Liver cancer	0
Vemurafenib	CRC	5			

^a^obtained from ClinivalTrials.gov

## Conclusion

FDA-approved anticancer medicines play important roles in the successful cancer treatment and novel anticancer drug development. In this study, we comprehensively collected 150 FDA-approved anticancer drugs from 1949 to 2014. According to their action mechanisms, we groups them into two sets: cytotoxic and targeted agency. Then we performed a comprehensive analysis from the perspective of drugs, drug indications, drug targets, and their relationships. For drugs, we summarized their historical characteristics and delivery methods. For targets, we surveyed their cellular location, functional classification, genetic patterns. We further applied network methodology to investigate their relationships. In this study, we provided a comprehensive data source, including anticancer drugs and their targets and performed a detailed analysis in term of historical tendency and networks. Its application to discover novel drug-cancer associations demonstrated that the data collected in this study is promising to serve as a fundamental for anticancer drug repurposing and development.

## Abbreviations

BLCA: Bladder urothelial carcinoma
BRCA: Breast adenocarcinoma
CLL: Chronic lymphocytic leukemia
COAD/READ: Colon and rectal adenocarcinoma
EGFR: Epidermal growth factor receptor
FDA: Food and drug administration
FL: Follicular B-cell non-Hodgkin lymphoma
GBM: Glioblastoma
HNSC: Head and neck squamous cell carcinoma
IPA: Ingenuity pathway analysis
KDR: Kinase insert domain receptor
KIRC: Kidney renal clear cell carcinoma
K-S: Kolmogorov-Smirnor
LAML: Acute myeloid leukemia
LUAD: Lung adenocarcinoma
LUSC: Lung squamous cell carcinoma
MOA: Mechanism of action
NCI: National cancer institute
OCGs: Oncogenes
OV: Ovarian cancer
SLL: Small lymphocytic lymphoma
TSGs: Tumor suppressor genes
UCEC: Uterine corpus endometrioid carcinoma
